# Effect of Gold Electronic State on the Catalytic Performance of Nano Gold Catalysts in *n*-Octanol Oxidation

**DOI:** 10.3390/nano10050880

**Published:** 2020-05-02

**Authors:** Ekaterina Pakrieva, Ekaterina Kolobova, Yulia Kotolevich, Laura Pascual, Sónia A. C. Carabineiro, Andrey N. Kharlanov, Daria Pichugina, Nadezhda Nikitina, Dmitrii German, Trino A. Zepeda Partida, Hugo J. Tiznado Vazquez, Mario H. Farías, Nina Bogdanchikova, Vicente Cortés Corberán, Alexey Pestryakov

**Affiliations:** 1 Research School of Chemistry & Applied Biomedical Sciences, National Research Tomsk Polytechnic University, Lenin Av. 30, 634050 Tomsk, Russia; ekaterina_kolobova@mail.ru (E.K.); germandmitry93@gmail.com (D.G.); 2 Instituto de Catálisis y Petroleoquímica, Consejo Superior de InvestigacionesCientíficas, Marie Curie 2, 28049 Madrid, Spain; laura.pascual@icp.csic.es (L.P.); vcortes@icp.csic.es (V.C.C.); 3 Centro de Nanociencias y Nanotecnología, Universidad Nacional Autónoma de México, P.O. Box 14, Ensenada 22800, Mexico; julia.kotolevich@gmail.com (Y.K.); trino@cnyn.unam.mx (T.A.Z.P.); tiznado@cnyn.unam.mx (H.J.T.V.); mario@cnyn.unam.mx (M.H.F.); nina@cnyn.unam.mx (N.B.); 4 LAQV-REQUIMTE, Department of Chemistry, NOVA School of Science and Technology, Universidade, NOVA de Lisboa, 2829-516 Caparica, Portugal; sonia.carabineiro@fct.unl.pt; 5 Department of Chemistry, Lomonosov Moscow State University, 1-3 Leninskiye Gory, GSP-1, 119991 Moscow, Russia; kharl@kge.msu.ru (A.N.K.); dashapi@mail.ru (D.P.); nnikitina1719@gmail.com (N.N.)

**Keywords:** gold catalysts, *n*-octanol oxidation, gold active sites, gold electronic state, support modifiers, metal content, pretreatment atmosphere, DFT, solvent adsorption, acid-base centers, selectivity

## Abstract

This study aims to identify the role of the various electronic states of gold in the catalytic behavior of Au/M_x_O_y_/TiO_2_ (where M_x_O_y_ are Fe_2_O_3_ or MgO) for the liquid phase oxidation of *n*-octanol, under mild conditions. For this purpose, Au/M_x_O_y_/TiO_2_ catalysts were prepared by deposition-precipitation with urea, varying the gold content (0.5 or 4 wt.%) and pretreatment conditions (H_2_ or O_2_), and characterized by low temperature nitrogen adsorption-desorption, X-ray powder diffraction (XRD), energy dispersive spectroscopy (EDX), scanning transmission electron microscopy-high angle annular dark field (STEM HAADF), diffuse reflectance Fourier transform infrared (DRIFT) spectroscopy of CO adsorption, temperature-programmable desorption (TPD) of ammonia and carbon dioxide, and X-ray photoelectron spectroscopy (XPS). Three states of gold were identified on the surface of the catalysts, Au^0^, Au^1+^ and Au^3+^, and their ratio determined the catalysts performance. Based on a comparison of catalytic and spectroscopic results, it may be concluded that Au^+^ was the active site state, while Au^0^ had negative effect, due to a partial blocking of Au^0^ by solvent. Au^3+^ also inhibited the oxidation process, due to the strong adsorption of the solvent and/or water formed during the reaction. Density functional theory (DFT) simulations confirmed these suggestions. The dependence of selectivity on the ratio of Brønsted acid centers to Brønsted basic centers was revealed.

## 1. Introduction

The selective oxidation of alcohols to valuable carbonyl compounds is one of the most crucial transformations in the chemical industry, for both laboratory and industrial manufacturing [1,2]. Traditionally, numerous oxidizing reagents (including toxic, expensive, stoichiometric oxidants [3,4]), along with the use of harmful solvents and harsh reaction conditions, have been employed to accomplish this transformation. Thus, these methods lead to environmental pollution and economic problems [5,6,7,8]. Therefore, there is considerable need for more harmless and sustainable technologies, requiring renewable feedstock, e.g., biomass, as replacement for fossil resources [9]. Therefore, a green process, involving the use of non-toxic and cheap oxygen, in the presence of a heterogeneous catalyst (used as a recyclable solid material in biomass processing), in mild conditions (atmospheric pressure, low temperature and absence of bases and radical initiators) would be of great interest [10,11,12,13].

Supported gold catalysts have proved to be efficient in the liquid phase oxidation of alcohols, due to their higher selectivity and better resistance to deactivation, compared to their conventional noble metal counterparts [14,15,16,17]. However, the effect of a number of parameters, such as gold content, particle size of gold, the preparation method, gold electronic state, influence of support modifiers, redox pretreatment, etc., have not been investigated in detail.

Notably, *n*-octanol belongs to the group of low fatty alcohols, whose physical properties impose constraints to the implementation of green chemistry approaches. One of the demanded oxidation product of *n*-octanol is octanal or caprylic aldehyde, which occurs naturally in citrus oils and could be used commercially as a component in perfumes and in flavor production for the food industry [18]. Compounds of octanoic acid, another *n*-octanol oxidation product, are found naturally in the milk of various mammals, and as a minor constituent of coconut and palm kernel oils [19]. Octanoic acid, also known as caprylic acid, has wide applications. In addition to the commercial production of ester (octyl octanoate) for perfumery, as flavor and fragrance agents, and in the manufacture of dyes, octanoic acid can be used as disinfectant [20] in commercial food handling and health care facilities. In addition, it is currently being investigated as a treatment for voice tremor [21,22] and taken as a dietary supplement [23].

Moreover, *n*-octanol is often used for comparative studies of catalytic activity in oxidation of alcohols, as a convenient model of primary alcohols of long chain. However, among them, there are very few studies where mild conditions have been applied for the efficient and selective liquid phase oxidation of *n*-octanol using gold supported catalysts; they are briefly summarized in Table 1.

Li et al. [24] studied silica-supported Au–Cu and Au–Ag alloy nanoparticles (NPs), for the aerobic oxidation of alcohols. For comparison, they also investigated the corresponding monometallic catalysts in octanol oxidation. With a large catalyst load, 6 wt.% Au/SiO_2_ (alcohol/Au ratio, R = 8), they achieved 40% conversion of *n*-octanol in 4 h, with 17% aldehyde selectivity. Unfortunately, no information of other products nor of electronic state of gold was provided.

Su et al. [25] suggested that the high dispersion of Au NPs and the electron donation effects of aryl rings to the Au NPs within the large cages of the MIL-101 support are the main reasons for the observed high activities of the Au/MIL-101 catalyst in the aerobic oxidation of alcohols, including *n*-octanol, under base-free conditions.

Liu et al. [26] showed that Au/γ-Ga_2_O_3_ was effective for the oxidation of several alcohols. In particular, 45% octanol conversion was obtained after 2.5 h with 99% aldehyde selectivity, using a low alcohol/Au ratio (R = 10). Nevertheless, the high catalytic performance exhibited was attributed to the significantly enhanced dehydrogenation capabilities, due to a strong interaction between Au NPs and the γ-Ga_2_O_3_ support, which was attributed to the presence of gold species detected by XPS with binding energy (BE) 83.1–83.4 eV.

Haider et al. [27] described the oxidation of various types of alcohols over 1 wt.% Au/Cu_a_Mg_b_Al_c_O_x_ catalysts. In the case of *n*-octanol oxidation, 34% octanal yield was obtained at 90 °C after 3 h (Table 1). Ex situ XANES of the fresh and spent catalysts revealed that a significant fraction of the deposited gold existed as charged species (Au^+^).

In a recent paper [28], MgO, ZnO and Nb_2_O_5_, representative of three different types of oxides (basic, amphoteric and acidic, respectively), were used as supports for Au NPs. It was found that the catalytic activity is influenced by the electron mobility between the Au NPs and the support, which depends on the intermediate electronegativity of the support. However, besides the predominant (Au^0^)*^δ^*^−^ species in some active dried catalysts (2 wt.% Au-MgO-D and 2 wt.% Au-ZnO-D), cationic gold (Au^+^) was also present. Selectivity in *n*-octanol oxidation, preferably towards ester formation in the case of the most active catalyst, was influenced by redox properties of the gold species, acid-base properties of the supports and catalyst pretreatment.

Our group widely investigated titania supported Au NPs with different modifiers, gold loading and treatments in *n*-octanol oxidation [29,30,31,32,33] (Table 1). It was found that monovalent gold ions are presumed to be the active sites in Au/M_x_O_y_/TiO_2_ (M = La or Ce) [31,32,33]. The concentration, strength and stability of these sites are determined by the gold content, the nature of the support and modifier, and the pretreatment atmosphere.

Modification and treatment in a hydrogen atmosphere of 4 wt.% Au/M_x_O_y_/TiO_2_ catalysts, where M_x_O_y_ = Fe_2_O_3_ or MgO modifiers, lead to the enhancement of Au/TiO_2_ catalyst activity in *n*-octanol oxidation [30]. Changes in the catalytic behavior were associated with a change in the electronic state of gold caused by the surface modification of TiO_2_ with oxides of magnesium and iron. However, the obtained experimental data turned out to be insufficient to identify the nature of the active site of these catalytic systems.

Therefore, in this work we aimed to study, in detail, how various electronic states of gold are formed under several factors, such as gold loading, treatment atmosphere, modifier nature, and how they affect the catalytic performance of Au/M_x_O_y_/TiO_2_ (M_x_O_y_ = Fe_2_O_3_ or MgO) catalysts in *n*-octanol oxidation under mild conditions.

## 2. Materials and Methods

Commercial nonporous TiO_2_ P25 (Evonik Degussa GmbH, Essen, Germany) was used as the initial support. Fe(NO_3_)_3_∙9H_2_O or Mg(NO_3_)_2_∙6H_2_O (Merck, Darmstadt, Germany) aqueous solutions were used for the modification of titania by impregnation method. Nominal molar ratio Ti/M (M = Fe or Mg) was 40. Impregnated supports were dried at room temperature for 48 h and at 110 °C for 4 h, with following calcination at 550 °C over 4 h. According to EDS results molar ratio Ti/M was closed to nominal ones (37–39) for both modified supports, and the ratio did not change significantly after gold deposition.

The method of deposition-precipitation with urea was used for gold deposition with 0.5 and 4 wt.% nominal loadings on the supports using HAuCl_4_∙3H_2_O (Merck, Darmstadt, Germany) as a precursor. The process of gold deposition was conducted in the absence of light, according to the previously reported procedure [34,35]. Shortly, the support was added in an aqueous solution (distilled water) containing 4.2 × 10^−3^ M of the gold precursor and 0.42 M urea (Merck, Darmstadt, Germany). This mixture had an initial pH of 2.4. After heating of solution at 80 °C for 16 h, the pH was adjusted to 7.5. The solution was then centrifuged at 11,000 rpm for 15 min and decanted, and the remaining solid washed with water; this procedure was repeated 4 times. The final stage was drying of samples under vacuum 2 h at 80 °C. Such samples before any pretreatment (H_2_ or O_2_ atmosphere) will be denoted herein as as-prepared samples. To prevent any alteration, the samples were stored in a desiccator under vacuum, away from light and at room temperature.

Nitrogen adsorption-desorption isotherms at −196 °C were used for the study of textural properties of catalysts and supports. Micromeritics TriStar 3000 apparatus (Norcross, GA, USA) was applied for the record of isotherms. Degassing of samples under vacuum at 300 °C for 5 h preceded each measurement. Normalization to standard temperature and pressure of adsorbed N_2_ volume was carried out. The BET method was applied to the nitrogen adsorption data (P/P_0_ range 0.05–0.25) for the calculation of the specific surface area (S_BET_) of the samples.

XRD patterns were obtained by using a Philips XPert PR diffractometer (Amsterdam, The Netherlands) using Ni-filtered CuKα (λ = 0.15406 nm) radiation. The step-scanning procedure included the following parameters (step size 0.02°; 0.5 s).

Scanning transmission electron microscopy-high angle annular dark field (STEM-HAADF) measurements were conducted with a microscope (JEOL JEM-2100F, JEOL Ltd., Tokyo, Japan) operated at 200 kV. Prior to study, samples were abraded to a fine powder state and then, a drop of the suspension was deposited on a lacey carbon coated copper grid. For each sample, at least ten representative images from the microscope were acquired, and at least 150 particles for each sample were taken into account for obtaining particle size distribution. Gold contents were measured by energy dispersive spectroscopy (XEDS) in the same system of the microscope equipped with an Oxford INCA X-sight system detector (Oxford Instruments, Abingdon, Oxfordshire, UK).

XPS was applied for the study of gold electronic state on catalyst surface with a SPECS GmbH (SPECS Surface Nano Analysis GmbH, Berlin, Germany) custom-made system using a PHOIBOS 150 WAL hemispherical analyzer and a non-monochromated X-ray source (Al Kα X-rays 1486.6 eV, 200 W). Detailed information was given in our previous papers [31,32].

Diffuse reflectance Fourier transform infrared spectroscopy (DRIFTS) measurements were performed on a Bruker EQUINOX 55/S FTIR spectrometer (Bruker Optik GmbH, Ettlingen, Germany) equipped with a homemade accessory. All DRIFTS CO spectra on the catalyst samples were recorded at 20 Torr pressure (5% accuracy measurement), at room temperature with a resolution of 4 cm^−1^. Typically, the powdered sample was loaded in a quartz ampoule with a window of CaF_2_. Prior to measurements, the samples were calcined at 100 °C under vacuum (10^−4^ Torr) for 1 h. Then, each catalyst was studied in three states: as-prepared, and after pretreatments either in H_2_ or in O_2_ at 300 °C for 1 h at 100 Torr, and then cooled down to room temperature. After that, hydrogen or oxygen was evacuated and CO adsorption (>99%) was carried out. The obtained DRIFTS data were presented in the form of Kubelka–Munk units (KMU). DRIFT CO spectra were obtained by subtracting the CO gas phase spectrum and the baseline was corrected. Pure supports did not exhibit bands of adsorbed CO in this region of the spectrum under the mentioned conditions.

NH_3_-temperature-programmable desorption (TPD) and CO_2_-TPD methods were applied to the study of acid and basic properties of the catalysts and corresponding supports on a “Chemosorb” chemisorption analyzer (Neosib, Novosibirsk, Russia), equipped with a thermal conductivity detector (TCD), which was calibrated with NH_3_ or CO_2_ prior to analysis. The starting desorption temperature in the case of CO_2_ was 25 °C, while TPD of ammonia started from 100 °C. Additionally, carrier gas in NH_3_-TPD was helium; in CO_2_-TPD was argon. Otherwise, the experimental procedure was the same in both cases, except for the listed differences in these methods. Prior to the measurements, the samples were treated at 300 °C under an inert atmosphere (He or Ar) for 1 h, to remove the impurities adsorbed on the surface. Then, the temperature was decreased to 100 °C or 25 °C, followed by saturation with NH_3_ or CO_2_ for 1 h and flushing with He or Ar for 1 h, to remove physisorbed ammonia or carbon dioxide. After that, the temperature was increased to 600 °C at a rate of 10 °C min^−1^, under an inert atmosphere. For a comparative analysis, NH_3_ and CO_2_ desorption profiles of catalysts are divided into three temperature ranges: 100 (25)–200 °C, 200–400 °C and 400–600 °C, and are assigned to weak, medium and strong acid or basic sites, respectively.

Before catalytic experiments of oxidation of *n*-octanol (Merck, ≥99%, HPLC grade), as-prepared samples were treated either in O_2_ atmosphere (herein denoted as *n%* Au/(M_x_O_y_)/TiO_2__pO_2_) or in H_2_ atmosphere (herein denoted as *n*% Au/(M_x_O_y_)/TiO_2__pH_2_) at 300 °C for 1 h, where *n* is the gold content in wt.% and M is the metal (Fe or Mg) of the modifier oxide.

The catalytic properties were studied at the temperature 80 °C under atmospheric conditions for 6 h in a semi-batch reactor, which is a four-necked round bottom flask equipped with reflux, oxygen feed, thermocouple and a septum cap. The appropriate amount of catalyst sample in *n*-octanol/Au ratio = 100 mol/mol was added to a flask with 0.1 M solution of *n*-octanol (25 mL) in *n*-heptane (Supelco, ≥99%, HPLC grade). Oxygen was bubbled through the reaction mixture with 30 mL/min rate. To monitor the reaction progress, small aliquots of the reacting mixture by using nylon syringe filters (pore 0.45 μm) were collected at 0.25, 0.5, 1,2,4 and 6 h, and then were analyzed in a Varian 450 gas chromatograph (Varian Inc, Palo Alto, CA, USA) with flame ionization detector (FID), using a capillary DB wax column (15 m × 0.548 mm, Varian Inc., Palo Alto, CA, USA), and He as the carrier gas. The attribution of peaks was made by comparison with chromatograms of genuine samples. In the absence of support/catalyst, no activity was observed in oxidation of *n*-octanol.

Alcohol conversion (*X*) and product yield (*Y_i_*) and selectivity (*S_i_*) were calculated in terms of moles of C atoms, as follows:

(1) $$Y_{i}=\frac{n_{i}C_{i}}{8\cdot C_{ROHin}}\cdot 100$$

where *n_i_* is the number of carbon atoms in compound *i*, and *C_i_* is its molar concentration, and *C_ROH_in* represents initial octanol concentration.

Conversion was calculated as the sum of the yields of carbon containing products, and product selectivity as the ratio between product yield and conversion.

(2) $$X_{ROH}=\sum Y_{i}$$

(3) $$S_{i}=\frac{Y_{i}}{X_{ROH}}\cdot 100$$

Carbon balances in all reported data were within 100 ± 3%.

The adsorption of *n*-octanol on gold nanoparticles was simulated using the density functional theory PBE [36] to determine at the atomic level the type of adsorption sites (Au^0^ or Au^+^, top atom or facet atom) of nanogold catalysts. As a model of nanoparticles, the tetrahedral Au_20_ cluster was considered. The experimentally observed Au_20_ cluster is 1 nm in size [37]. Because the cluster has atoms with different coordination numbers (on the top, on the facet and on the edge), it has been a popular model for studying the structural effects in adsorption and catalysis [38,39].

We simulated the *n*-octanol adsorption as a reaction:

Au_20_ + C_7_H_15_CH_2_OH → (C_7_H_15_CH_2_OH)Au_20_ (R1)

Au_20_^+^ + C_7_H_15_CH_2_OH → (C_7_H_15_CH_2_OH)Au_20_^+^ (R2)

The different coordination of alcohol on Au_20_ was considered. The structures of (C_7_H_15_CH_2_OH)Au_20_ were optimized, and the total energies of the reagents and products were calculated, taking into account the energy of zero vibrations. The change in total energy and standard enthalpies of the Reactions (R1) and (R2) at the 100 °C temperature were determined according to the formulas:

ΔE_1_ = E(octanol-Au_20_) − E(Au_20_) − E(octanol) (4)

ΔE_2_ = E(octanol-Au_20_^+^) − E(Au_20_^+^) −E(octanol) (5)

To reveal the role of different gold sites in the adsorption of solvent molecules, heptane adsorption on simple (Au^0^O)^2−^, (Au^+^O)^−^, and (Au^3+^O)^+^ models containing Au^0^, Au^+^, and Au^3+^ was also studied at the atomic level using density functional theory calculation with PBE functional [36]. We simulated C_7_H_16_ adsorption through Reactions (R3)–(R5):

(Au^0^O)^2−^ + C_7_H_16_ → C_7_H_16_-(Au^0^O)^2−^ (R3)

(Au^+^O)^−^ + C_7_H_16_ → C_7_H_16_-(Au^+^O)^−^ (R4)

(Au^3+^O)^+^+ C_7_H_16_ → C_7_H_16_-(Au^3+^O)^+^ (R5)

The structures of all molecules were fully optimized, and the total energies of the reagents and products were calculated taking into account the energy of zero vibrations. Adsorption energies were calculated as the difference in total energies of adsorbed complex and the reagents (heptane and (Au^0^O)^2−^/(Au^+^O)^−^/(Au^3+^O)^+^).

All density functional theory (DFT) calculations were performed in the PRIRODA program (version 17, Russia) [40], using a Lomonosov supercomputer [41].

## 3. Results and Discussion

### 3.1. Catalytic Results

The results showed that the gold content, support nature and the pretreatment atmosphere significantly affected the catalytic properties of gold catalysts in the liquid phase oxidation of *n*-octanol. The activity of catalysts in the as-prepared state was insignificant and practically does not depend on the nature of the support and gold content. The reason for this is that gold in as-prepared samples is found on the support surface in the form of a trivalent gold complex with urea hydrolysis products, which is catalytically inactive [30,42].

**Figure 1:**
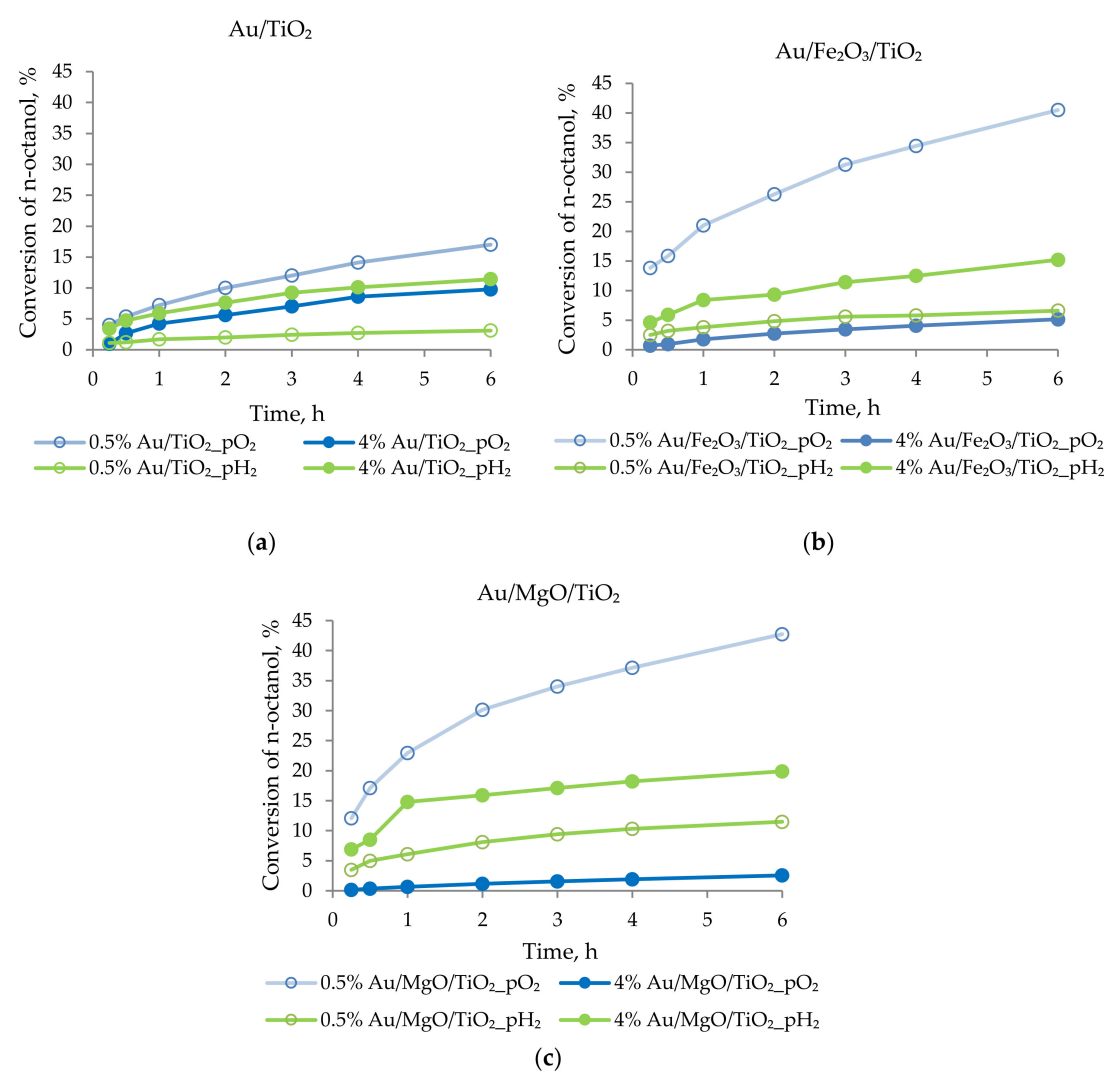
Effect of gold loading and pretreatments on the oxidation of *n*-octanol on Au/M_x_O_y_/TiO_2_ (M_x_O_y_ = Fe_2_O_3_, MgO) catalysts: evolution of conversion with run time (**a**–**c**).

However, for most of the studied catalysts, the activity increased several times after either the reduction or oxidation treatment. However, depending on the pretreatment atmosphere, the effect of gold content on activity was different (Figure 1). After H_2_ treatment, the order of activity was: Au/MgO/TiO_2_ > Au/Fe_2_O_3_/TiO_2_ > Au/TiO_2_, and activity increased with an increase of gold content in the three cases (Figure 1).

**Figure 2:**
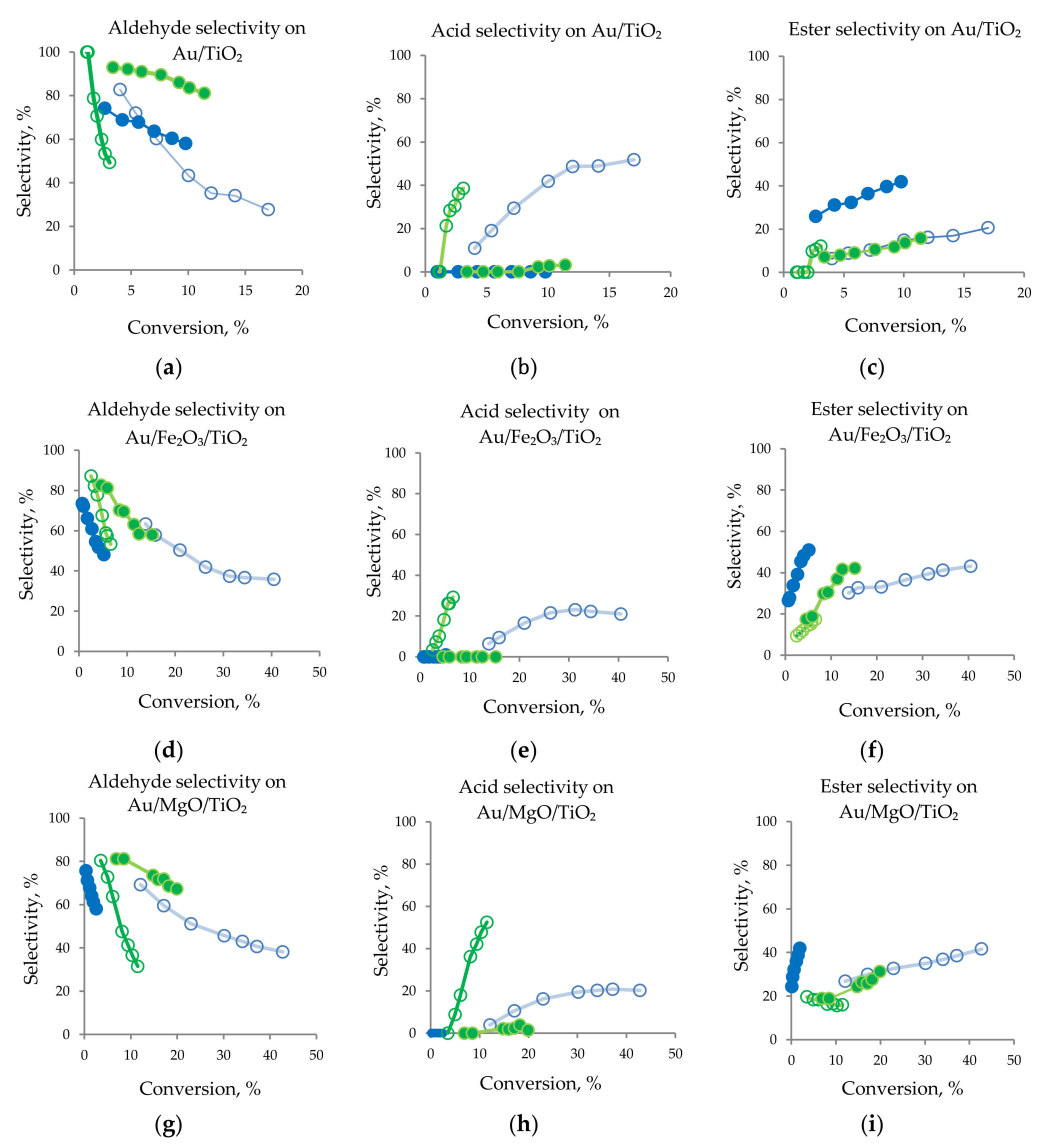
Effect of gold loading and pretreatments on the oxidation of *n*-octanol on Au/M_x_O_y_/TiO_2_ (M_x_O_y_ = Fe_2_O_3_, MgO) catalysts. Products selectivity evolution with conversion for Au/TiO_2_ (**a**–**c**), Au/Fe_2_O_3_/TiO_2_ (**d**–**f**) and Au/MgO/TiO_2_ (**g**–**i**) is shown. Green lines: results after hydrogen pretreatment, blue lines: after oxygen pretreatment. Catalysts Au contents: hollow symbols show catalysts with 0.5% Au; full symbols with 4% Au.

Analysis of the products distribution (Figure 2) for 0.5% Au/TiO_2__pH_2_, 0.5% Au/Fe_2_O_3_/TiO_2__pH_2_, 0.5% Au/MgO/TiO_2__pH_2_ samples showed that selectivity to acid formation (39%, 30% and 53% respectively) increased with run time, while selectivity to aldehyde sharply decreased from 100% at the reaction start down to 50% for 0.5% Au/TiO_2__pH_2_, 53% for 0.5% Au/Fe_2_O_3_/TiO_2__pH_2_ and 30% for 0.5% Au/MgO/TiO_2__pH_2_. Ester formation was at 12% and 17% levels for unmodified sample and modified samples, respectively. The selectivity trends were totally different for their homologues with 4 wt.% gold loading after hydrogen pretreatment. The main product was octanal. Octanoic acid formation was practically negligible; only some traces of acid were detected at longer run times for 4% Au/TiO_2__pH_2_ and 4% Au/MgO/TiO_2__pH_2_. For 4% Au/Fe_2_O_3_/TiO_2__pH_2_; selectivity towards octyl octanoate increased at the expense of octanal formation with no acid formation.

**Scheme 1:**
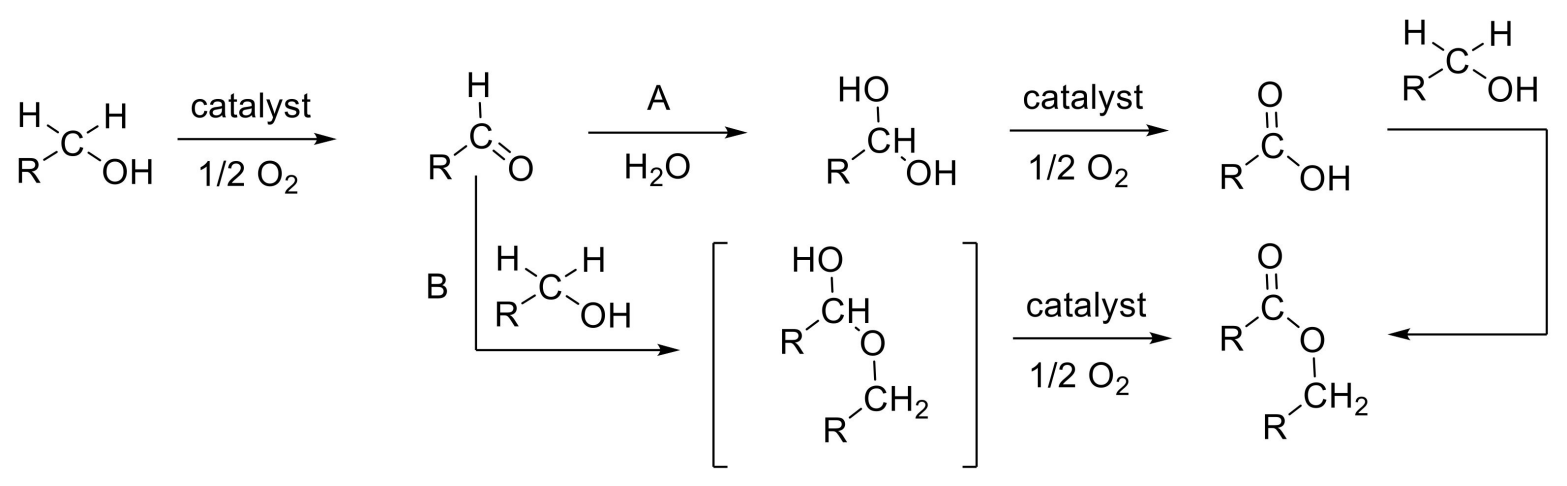
Possible reaction pathways for the oxidation of *n*-octanol on supported gold catalysts (adapted from [29,30,31,32,33,43]).

Such changes in the reaction product distribution with an increase in Au content should be caused by alteration in the acid-base properties of support. Aldehyde formation requires only the first step of the reaction mechanism, that occurs on the gold active surface, while the formation of octanoic acid and octyl octanoate requires a second mechanistic step [29,30] through intermediates formed by acid-base catalyzed reactions, proceeding mainly on the support surface. In addition, in the scheme proposed by several authors [29,30,31,32,33,43], the formation of ester or acid can occur by two routes: hydration to a geminal diol (Scheme 1, A) that can be further oxidized to octanoic acid, or an acetalization to form the hemiacetal, that can be oxidized to octyl octanoate. The ester could also be formed by the esterification of the acid with the alcohol (Scheme 1, B).

In contrast, after pretreatment of samples in an oxygen atmosphere, the opposite dependence on the activity of the gold content was observed, i.e., the catalytic activity decreased with the increase of gold content for all supports. Similarly good results were achieved using modified samples with the lower Au content after oxidation pretreatment at 300 °C for 1 h, namely 0.5% Au/MgO/TiO_2__pO_2_ and 0.5% Au/Fe_2_O_3_/TiO_2__pO_2_: the conversion of *n*-octanol after 6 h reached 43% and 41%, respectively, with increasing ester selectivity in both cases (Figure 1 and Figure 2). Acid formation (up to 52% selectivity) was observed only for the unmodified catalyst, 0.5% Au/TiO_2__pO_2_. In case of modified samples, acid formation decreased after 3 h of reaction and was 20% after 6 h for both catalysts.

For catalysts with higher gold loading after oxygen treatment, similar trends in selectivity can be seen for samples with samples after hydrogen treatment: almost complete absence of acid (1% of acid was found only for 4% Au/Fe_2_O_3_/TiO_2__pO_2_). However, the difference is a larger formation of ester: 20% in case of 4% Au/TiO_2__pO_2_, 40% and 42%, in case of 4% Au/MgO/TiO_2__pO_2_ and 4% Au/Fe_2_O_3_/TiO_2__pO_2_, respectively. At the same time, it is worth noting that the activity of these catalysts was very low, especially for modified samples (Figure 1b,c).

A common feature of catalysts with 0.5% Au, independently of the pretreatment, was the preferential formation of acid, but a larger tendency to ester formation was found for catalysts with oxygen pretreatment; also 0.5% Au/MgO/TiO_2_ and 0.5% Au/Fe_2_O_3_/TiO_2_ catalysts showed almost identical activity and selectivity in *n*-octanol oxidation.

### 3.2. Catalysts Characterization

To understand the differences observed in the catalytic behavior of the samples, a series of physicochemical studies were carried out.

**Figure 3:**
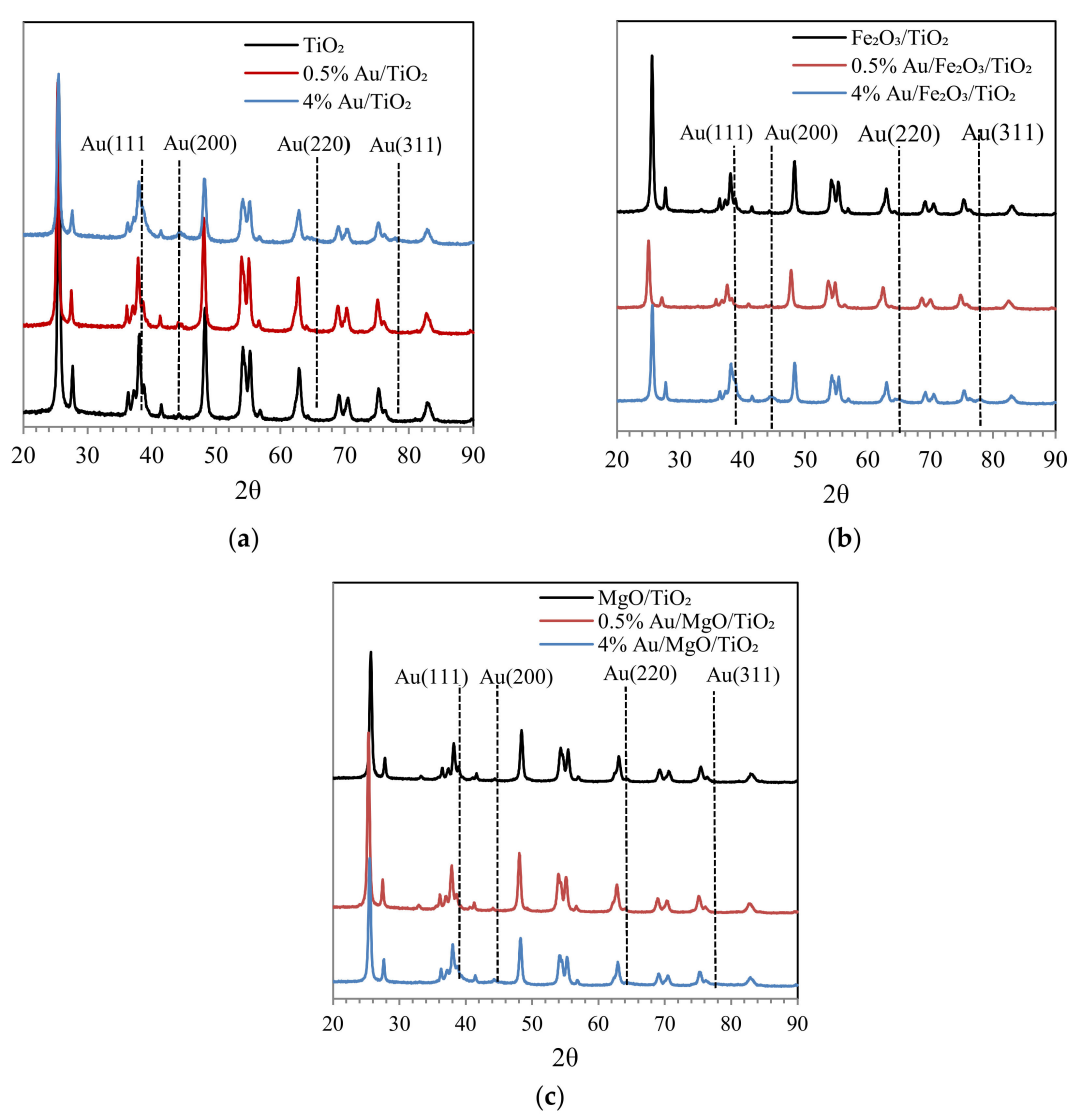
XRD patterns for catalysts Au/TiO_2_ (**a**), Au/Fe_2_O_3_/TiO_2_ (**b**), Au/MgO/TiO_2_ (**c**) treated in H_2_ (300 °C, 1 h) and their corresponding supports.

The XRD method was used to study the phase composition of the catalysts. The diffractograms (Figure 3) showed the absence of diffraction lines related to gold or the modifiers, indicating either small sizes of Au particles and metal oxides (lower than the XRD sensitivity threshold of 3–4 nm) [44] or their X-ray amorphous structure.

Table 2 summarizes the specific surface area (S_BET_) and gold content of the catalysts and their corresponding supports. Because of modification with both Fe and Mg oxides, S_BET_ of the initial TiO_2_ support was reduced by 13% (48–49 m^2^/g). Further gold deposition did not significantly change the supports’ S_BET_, except for catalysts with 4% Au, where S_BET_ had a 10% decrease. Elemental analysis showed that the actual gold loadings obtained were close to the nominal value.

**Figure 4:**
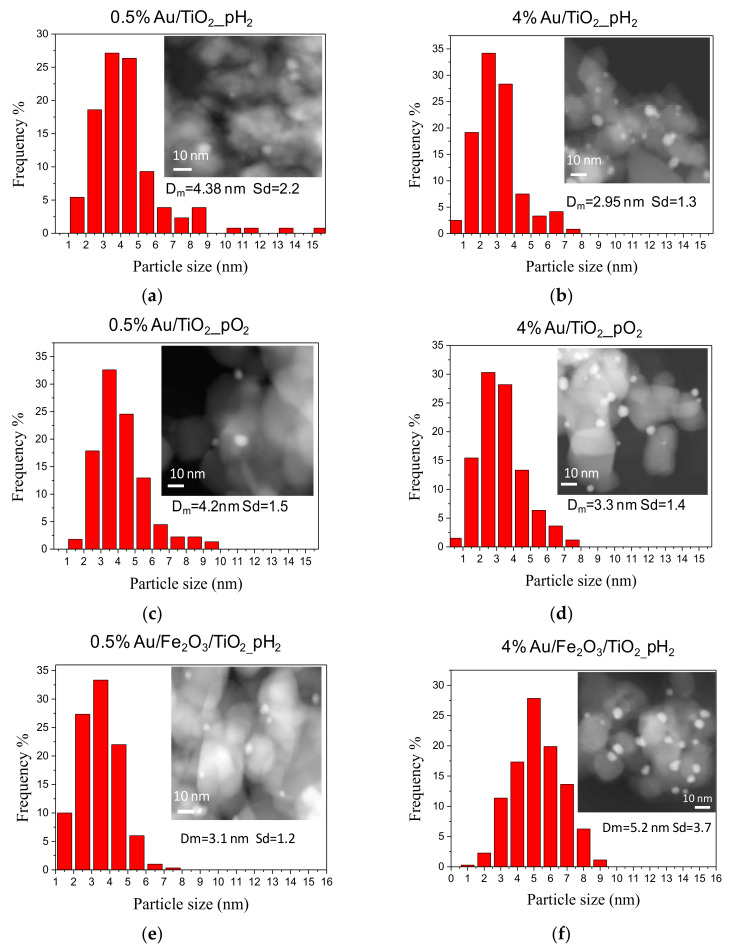

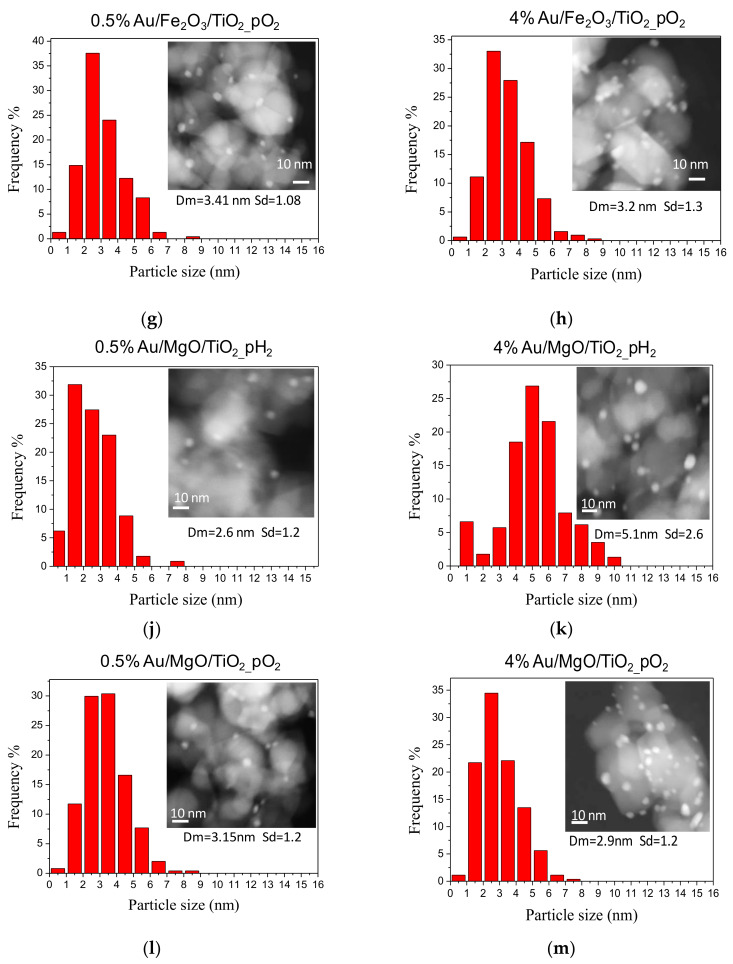
Au particle size distribution and representative STEM HAADF micrographs of catalysts Au/TiO_2_ (**a**–**d**), Au/Fe_2_O_3_/TiO_2_ (**e**–**h**), Au/MgO/TiO_2_ (**j**–**m**), for different gold amounts (0.5 or 4 wt.%) and pretreatment atmospheres (H_2_ or O_2_).

Table 2 also depicts the Au particle size obtained from STEM HAADF images presented in Figure 4. The distribution of gold nanoparticles for all the studied catalysts is in the range of 1–10 nm, with the exception of 0.5% Au/TiO_2__pH_2_, for which larger particles were observed; up to 15 nm (Figure 4a). The average size of Au NPs was found within the 2.6–5.2 nm interval for the studied materials (Table 2). The most active samples, namely 0.5% Au/Fe_2_O_3_/TiO_2__pO_2_ and 0.5% Au/MgO/TiO_2__pO_2_, with almost equal conversion (41% and 43%) had similar mean particle size (3.4 and 3.2 nm), respectively. However, catalysts with smaller particle sizes, like 0.5% Au/MgO/TiO_2__pH_2_ (2.6 nm) and 4% Au/MgO/TiO_2__pO_2_ (2.9 nm), demonstrated low activity in *n*-octanol oxidation (12% and 3% conversion, respectively). Therefore, taking into account the data from catalytic studies, no direct correlation between Au NPs average size and catalytic performance can be found.

**Figure 5:**
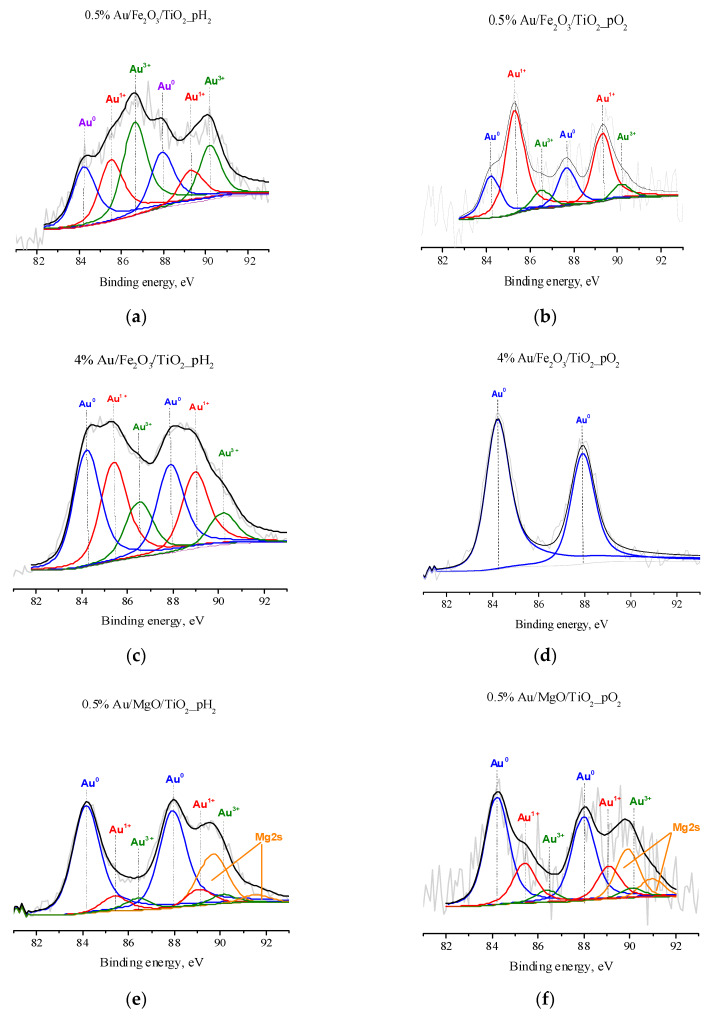

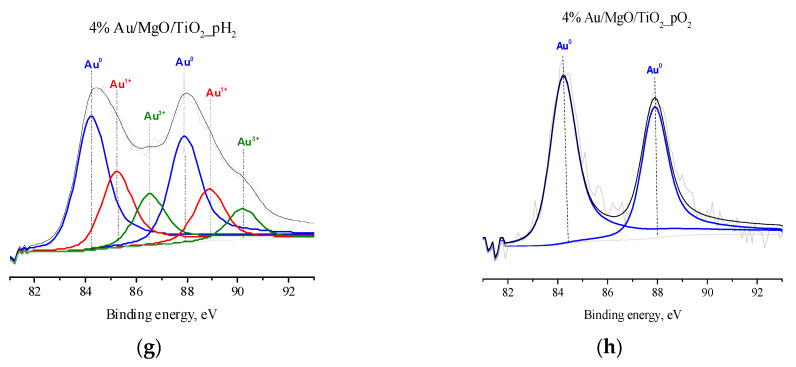
Au4f XP spectra of Au/Fe_2_O_3_/TiO_2_ and Au/MgO/TiO_2_ samples with different gold contents (0.5 or 4 wt.%), pretreated in H_2_ or O_2_ flow, at 300 °C, for 1 h: 0.5% Au/Fe_2_O_3_/TiO_2__pH_2_ (**a**), 0.5% Au/Fe_2_O_3_/TiO_2__pO_2_ (**b**), 4% Au/Fe_2_O_3_/TiO_2__pH_2_ (**c**), 4% Au/Fe_2_O_3_/TiO_2__pO_2_ (**d**), 0.5%Au/MgO/TiO_2__pH_2_ (**e**), 0.5% Au/MgO/TiO_2__pO_2_ (**f**), 4% Au/MgO/TiO_2__pH_2_ (**g**), 4% Au/MgO/TiO_2__pO_2_ (**h**).

**Figure 6:**
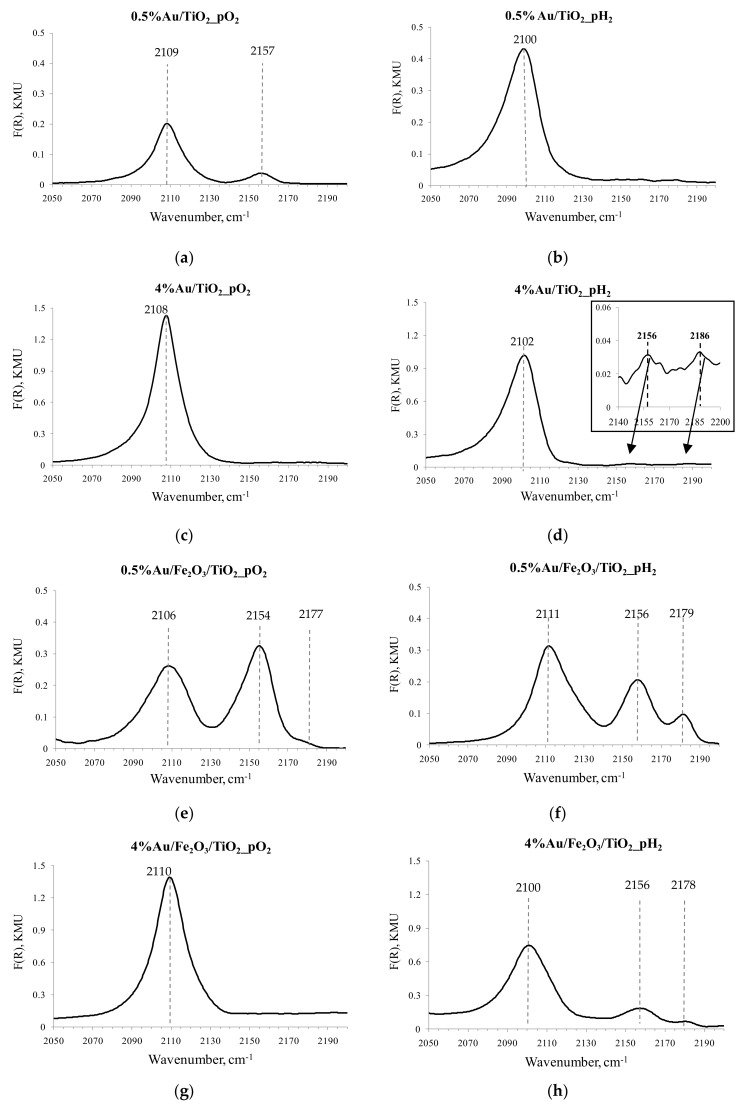

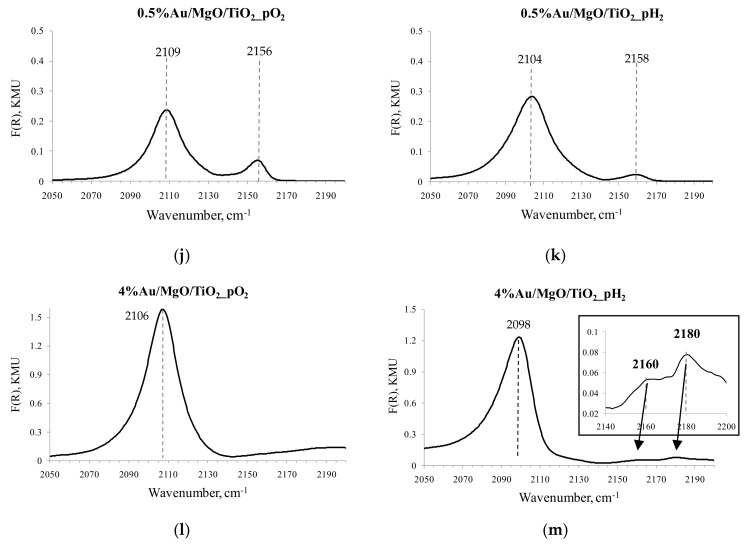
DRIFT spectra of CO adsorbed on catalysts pretreated at 300 °C for 1 h under a H_2_ or O_2_ atmosphere.

Nevertheless, one should note that according to DRIFT CO studies and XPS presented below (Table 3, Figure 5 and Figure 6), there is a fraction of gold in ionic state (Au^+^ or Au^3+^), which is not detected by STEM. The amount of these gold species depends on the support, pretreatment and gold content. Furthermore, for such a type of reaction, not all metal particles visible on the micrographs are active participants in the catalytic process; in some cases, only particles with a size below the threshold of the technique (1 nm) and less are active, as reported in the literature and in our previous works [29,30,45,46,47].

The XPS method was applied to investigate gold electronic states onthe support surface. The binding energies of Au 4f spectra according to XPS measurements show various states of gold, ions Au^+^ and Au^3+^ and neutral gold, which were affected by the support composition, pretreatment atmosphere, and Au content (Table 3). The XPS spectra for modified samples are presented in Figure 5.

All samples showed BE (Au4f_7/2_) 84.2 eV (26–100% relative content), attributed to Au^0^ states [48,49,50,51,52]; this metallic gold was the only state found for 4% Au/MgO/TiO_2__pO_2_ and 4% Au/Fe_2_O_3_/TiO_2__pO_2_. Besides the peak due to the metallic gold, another Au4f_7/2_ peak in the BE range 85.2–85.5 eV, attributed to single charged ions (Au^+^) [49,50,51,52], was detected in all the other catalysts, with 9–64% relative content. The highest amount of Au^+^ was found in the modified samples, with 0.5% Au after oxygen treatment and 4% Au after hydrogen treatment (Table 3, Entries 6, 7, 10 and 11). The 0.5% Au/Fe_2_O_3_/TiO_2__pO_2_ sample showed the maximum contribution of single charged ions (64%), compared with other catalysts.

Moreover, another oxidized state of gold, related to three-charged gold (Au^3+^) with BE in the range 86.4–86.6 eV (Au4f_7/2_) [53,54,55] was found for 4% Au/TiO_2__pH_2_ [31], and in modified catalysts with different contents (8–43%), the highest portion of Au^3+^ being found for 0.5% Au/Fe_2_O_3_/TiO_2__pH_2_.

It should be noted that in both 0.5% Au/MgO/TiO_2__pH_2_ and 0.5% Au/MgO/TiO_2__pO_2_ catalysts, there was an overlapping of Au4f_7/2_ line with the Mg2s line (Figure 5e,f), leading to a complication of these peaks’ interpretation, with a subsequent uncertainty. However, since the Au4f_7/2_ line is clearly visible, the states are identified correctly.

In order to obtain more information on the gold electronic states of the catalysts, DRIFT spectroscopy of CO adsorption was used. As can be seen from Figure 6, an absorption band with the maximum in the range of 2090–2130 cm^−1^, corresponding to the surface carbonyl groups on gold atoms Au^0^–CO [56], was observed for all catalysts. The different intensities of the absorption bands corresponding to Au^0^–CO can be explained by carbon monoxide being very weakly adsorbed on metallic gold, due to some features of the σ-π bond in M^0^–CO for Au compared to other noble metals [57]. Therefore, only the highly dispersed clusters or gold atoms can be sites for the adsorption of CO.

Comparing the data on the band intensity related to Au^0^–CO with the STEM results, it can be seen that the average particle size for samples with low frequency absorption bands is larger than for samples with high band intensity, and the differences in signal position are due to the adsorption of CO on metal clusters of different sizes. In fact, considering the DRIFT CO results for samples with the same support and Au content but different pretreatment, e.g., 4% Au/MgO/TiO_2__pO_2_ (Figure 6l) and 4% Au/MgO/TiO_2__pH_2_ (Figure 6m), a higher intensity of the Au^0^–CO band is observed for the latter, meaning that this catalyst should have smaller particles than the former. This is confirmed by the STEM results shown in Table 2: Entry 7 (4% Au/MgO/TiO_2__pO_2_) 5.1 nm and Entry 8 (4% Au/MgO/TiO_2__pH_2_) 2.9 nm. The same correlation between the average particle size and the intensity of the absorption band of metal particles was observed for the rest of the catalysts.

Another absorption band with the maximum in the range 2150–2170 cm^−1^, related to the complexes of ions Au^+^–CO [58,59], was observed in all cases, except for 4% Au/Fe_2_O_3_/TiO_2__pO_2_ and 4% Au/MgO/TiO_2__pO_2_ (Figure 6c,g,l). These results are in a good agreement with XPS data, as no Au^+^ was detected in these catalysts (Figure 5d,h). This absorption band is less intense than the one attributed to Au^0^–CO, except for 0.5% Au/Fe_2_O_3_/TiO_2__pO_2_ (Figure 6e), which correlates well with the predominant content of Au^+^ (64%) found by XPS in this catalyst (Table 3, Entry 10).

Along with absorption bands at 2090–2130 cm^−1^ and 2150–2170 cm^−1^, ascribed to carbonyls of metallic and singly charged gold, respectively, a third band appears within 2170–2190 cm^−1^ range for 4% Au/TiO_2__pH_2_, 4% Au/Fe_2_O_3_/TiO_2__pH_2_, 4% Au/MgO/TiO_2__pH_2_, 0.5% Au/Fe_2_O_3_/TiO_2__pH_2_ and 0.5% Au/Fe_2_O_3_/TiO_2__pO_2_ catalysts, its interpretation being ambiguous. In most studies, this absorption band is assigned to the adsorption of CO on monovalent gold ions, as it is believed that a higher-charged gold cation (Au^3+^) is very unstable, or even does not form carbonyl species. That could be caused by the following reasons: (1) Au^3+^ ions are very easily reduced with CO [60,61,62]; (2) since Au^3+^ ions are strongly charged, probably, trivalent ions on a support surface are usually saturated by coordination, and the evacuation at elevated temperatures will easily lead to the reduction of Au^3+^ [63].

At the same time, according to XPS (Table 3 and Figure 5), Au^3+^ was found for 4% Au/TiO_2__pH_2_, 4% Au/Fe_2_O_3_/TiO_2__pH_2_, 4% Au/MgO/TiO_2__pH_2_ samples, even after the reduction treatment at 300 °C. Moreover, Au^3+^ relative content in these samples is much higher than in the oxidized ones. Luengnaruemitchai et al. [55] have observed that Au^3+^ remains in Au/Fe_2_O_3_/TiO_2_ catalytic systems, even after calcination at 400 °C. Such trivalent cations could be stabilized by oxygen vacancies, formed under the action of high temperature reduction treatment during the preparation of the catalyst [64,65,66,67]. It is also worth noting that the reducibility of supports such as titania increases after the gold deposition, due to metal-support interaction. A similar mechanism for the stabilization of gold ions was proposed elsewhere [68]. In addition, it was also suggested that modification of the titania surface leads to an increase in the number of oxygen defects, which in turn increases the number of stabilized gold ions. A similar trend was observed in our study.

Mihaylov et al. [63] carried out CO adsorption IR experiments for the characterization of supported gold catalysts in conditions comparable to our study, and proved that the absorption band with a maximum in the range of 2170–2190 cm^−1^ corresponds to trivalent gold in non-exchange positions. Thus, we can also assume that the absorption band within 2176–2186 cm^−1^ interval belongs to the trivalent gold ion. Additionally, this is in good agreement with the XPS data.

Because no direct correlation was found between the average particle size of gold and the catalytic activity of samples, and neither significant differences in the texture and structural properties of the studied catalysts were found, it can be assumed that these parameters are not determining for the observed catalytic behavior of our systems. However, another parameter capable of influencing the catalytic behavior of gold catalysts is its electronic state. Based on the analysis performed on the surface of the studied catalysts by the above methods (XPS, DRIFT CO), three electronic states of gold were found, with different relative concentrations and ratios.

Table 3 shows the relative content of the electronic states of gold found in the samples and *n*-octanol conversion after 6 h. It can be seen that there is no relationship between the conversion and the content of gold in cationic states (Au^+^ and Au^3+^), if each state is considered separately. However, if the combined concentration of Au^+^ and Au^3+^ is considered, one tendency can be seen: for samples with a higher concentration of Au^+^, but with a lower contribution of Au^3+^, the conversion of octanol is higher. For example, for 0.5% Au/MgO/TiO_2__pH_2_ and 0.5% Au/MgO/TiO_2__pO_2_ (Table 3, Entries 5 and 6, respectively), the Au^3+^ content is the same (8%). However, the sample after oxygen pretreatment has a larger contribution of monovalent gold (25%) than the reduced sample (11%); similarly, the conversion for Au/MgO/TiO_2__pO_2_ (43%) sample is much higher than for 0.5% Au/MgO/TiO_2__pH_2_ (12%). Furthermore, for the same content of monovalent ions (29%), in 4% Au/MgO/TiO_2__pH_2_ and 0.5% Au/Fe_2_O_3_/TiO_2__pH_2_ catalysts (Table 3, Entries 7 and 9, respectively), the octanol conversion is higher in the latter sample, where the content of trivalent gold is 23% lower than in the former. It is worth noting that for samples with a high content of metallic gold (Table 3, Entries 8 and 12), the conversion of *n*-octanol is very low, 3 and 5%, respectively.

Therefore, by comparing the spectroscopic and catalytic results, it can be assumed that monovalent gold ions (Au^+^) are probably responsible for enhanced catalytic activity, while metallic gold (Au^0^) and three-charged gold (Au^3+^) have a negative effect on the catalytic activity. The DFT calculations of *n*-octanol adsorption on tetrahedral gold clusters presented in Section 3.4 below proved that monovalent gold ions play an important role in the *n*-octanol oxidation.

The negative effect of metallic and trivalent gold on catalytic activity probably occurs due to the strong adsorption of the solvent (*n*-heptane) on both Au^0^ and Au^3+^ or water generated during the dehydrogenation reaction of *n*-octanol on Au^3+^ (Scheme 1). The calculations of the adsorption energy in Section 3.5 below confirmed this assumption.

### 3.3. Study of Acid-base Properties of Catalysts

CO_2_-TPD and NH_3_-TPD methods were used to reveal which centers on the surface of the supports are responsible for the secondary reactions in the oxidation of *n*-octanol; namely, the formation of ester or acid. Three types of acid and basic sites with different concentration and strength (weak, medium and strong), depending on the temperature range where CO_2_ or NH_3_ desorption occurs, reflecting their nature, were detected for studied catalysts (Table 4 and Table 5). Both basic and acid centers with weak (25–200 or 100–200 °C) and medium strength (200–400 °C) are usually associated with surface hydroxyl groups, i.e., the Brønsted basic centers (BBC) and Brønsted acid centers (BAC). Strong acid centers (400–600 °C), along with protonated sites (hydroxyl groups), can also have aprotic nature and represent Lewis acid centers (LAC), which could include cations of gold, titanium or modifiers. Strong basic centers (400–600 °C) are associated with low-coordinated oxygen anions.

As known, esterification of alcohols is catalyzed by H^+^. On the catalyst surface, most likely, the Brønsted acid centers act as the source of H^+^. At the same time, the formation of acid requires the presence of water, which on the catalyst surface can be in the form of pairs of Brønsted basic and acid centers (BBC and BAC), not excluding adsorbed water, which in turn, can dissociate on the catalyst surface with the formation of a pair of BBC and BAC. Accordingly, both acid and basic Brønsted centers must be present on the surface of the catalyst for the reaction to proceed along route A, including the esterification reaction (Scheme 1). In turn, BAC should mainly be on the surface of the catalyst for the reaction to proceed along route B.

In relation to the studied catalysts, a direct correlation between the acid-base properties determined by the TPD of NH_3_ and CO_2_ and the selectivity for acid or ester was not found (Table 6), which is most likely due to a combination of a number of reasons; namely, different activity of the catalysts, the presence of trivalent gold and metallic gold (due to the adsorption of the solvent on trivalent gold and metallic gold not only the deactivation of the catalyst occurs, but also a change in selectivity, which leads to disconformities between the acid-base properties and ester and/or acid selectivity), as well as some features of desorption methods for determining acid-base properties, namely TPD of NH_3_ and CO_2_, described next.

Firstly, one should take into account the differences in temperatures: in this case, the oxidation of *n*-octanol tests (T = 80 °C), and the temperature ranges of the TPD study (CO_2_ desorption starts from 25 °C, meanwhile NH_3_ desorption starts from 100 °C to exclude the contribution of ammonia’s physical adsorption). It should be noted also that mainly due to this reason, we take into account only the concentrations of weak acid and basic centers. It is also well known that strong BAC and BBC are active participants in the catalytic process only at elevated temperatures (high-temperature gas-phase reactions).

Secondly, the probe molecules can change the chemical properties of the surface due to chemical transformations. Such examples are: ammonia dissociation at elevated temperatures on NH_2_^−^ and H^+^, giving false centers [69]; in addition, as shown in [70,71], a carbon dioxide molecule adsorbed on small gold nanoparticles can react with gold at room temperature (oxidizing metallic gold: 2Au^0^ + CO_2_ → Au^+^–O_2_–Au^+^ + CO) and, accordingly, provide new basic sites, not directly related to the catalyst.

Thirdly, the probe molecules do not coincide in size with the reagent molecules and there will always be the issue of comparing the results of measuring acidity (basicity) and catalytic behavior.

Fourthly, the TPD methods of ammonia and CO_2_ give information only about the strength and concentration of acid and basic centers, but not about their nature (Brønsted acid centers, Brønsted basic centers, Lewis acid centers and Lewis basic centers).

Nevertheless, for most of the studied systems, with the exception of samples with a high content of trivalent gold, there is a general tendency in the selectivity for acid and/or ester, depending on the acid-base properties of the catalysts (Table 6), namely:

For 0.5% Au/TiO_2__H_2_ and 0.5% Au/TiO_2__O_2_ samples (Table 6, Entries 1 and 2) with a content of weak acid (100 and 167 μmol/g) and basic (46 and 56 μmol/g) centers, with a ratio of 2:1 and 3:1, respectively, acid and ester were observed in the reaction products, with the predominant formation of acid (the reaction proceeds along route A, Scheme 1).

For samples 4% Au/TiO_2__O_2_, 4% Au/Fe_2_O_3_/TiO_2__pO_2_, 4% Au/MgO/TiO_2__pO_2_ and 4% Au/TiO_2__pH_2_ (Table 6, Entries 4, 8, 12 and 3 respectively), with a significant predominance of BAC compared to BBC, the acid formation does not exceed 3%, and we can assume that the reaction proceeds mainly along route B, taking into account the low conversion of *n*-octanol for these samples.

When comparing 0.5% Au/Fe_2_O_3_/TiO_2__pO_2_ and 0.5% Au/MgO/TiO_2__pO_2_ (Table 6, Entries 6 and 10) samples with a similar conversion level and roughly the same distribution of reaction products, we can also note that the concentrations of acidic and basic centers for these samples are close, the ratio of BAC/BBC = 4.6 and 5.1, respectively.

It should be noted that with an increase in the ratio of BAC/BBC, ester begins to prevail in the reaction products. This is clearly seen when comparing the corresponding values of BAC/BBC and the selectivity for ester and acid for samples 0.5% Au/TiO_2__H_2_ and 0.5% Au/TiO_2__O_2_ (BAC/BBC ≤ 3), 0.5% Au/Fe_2_O_3_/TiO_2__pO_2_ and 0.5% Au/MgO/TiO_2__pO_2_ (BAC/BBC > 4.5) and 4% Au/TiO_2__O_2_, 4% Au/Fe_2_O_3_/TiO_2__pO_2_, 4% Au/MgO/TiO_2__pO_2_ and 4% Au/TiO_2__pH_2_ (BAC/BBC > 7.5).

### 3.4. Quantum Chemical Simulation of n-Octanol Adsorption on Tetrahedral Gold Cluster

**Figure 7:**
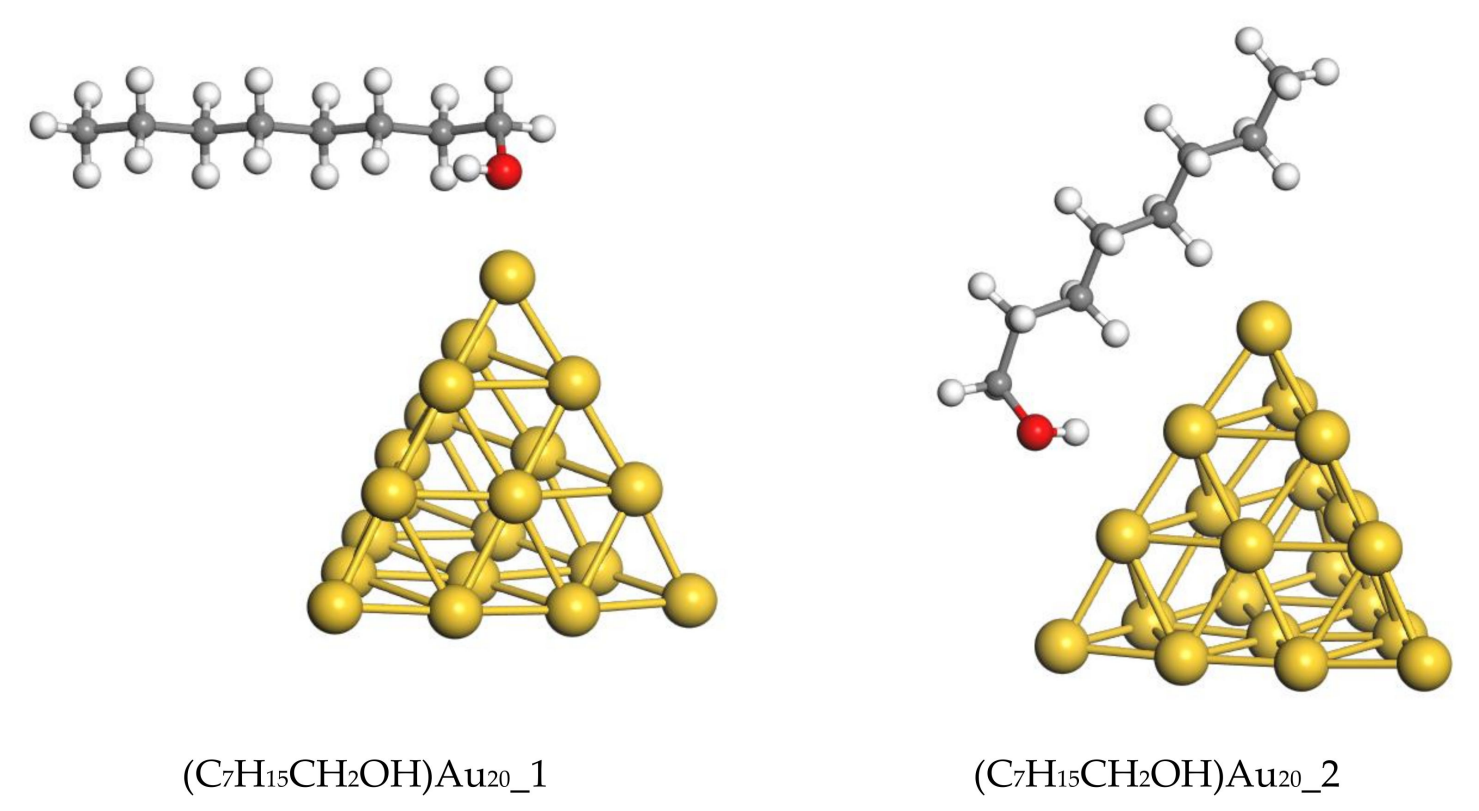

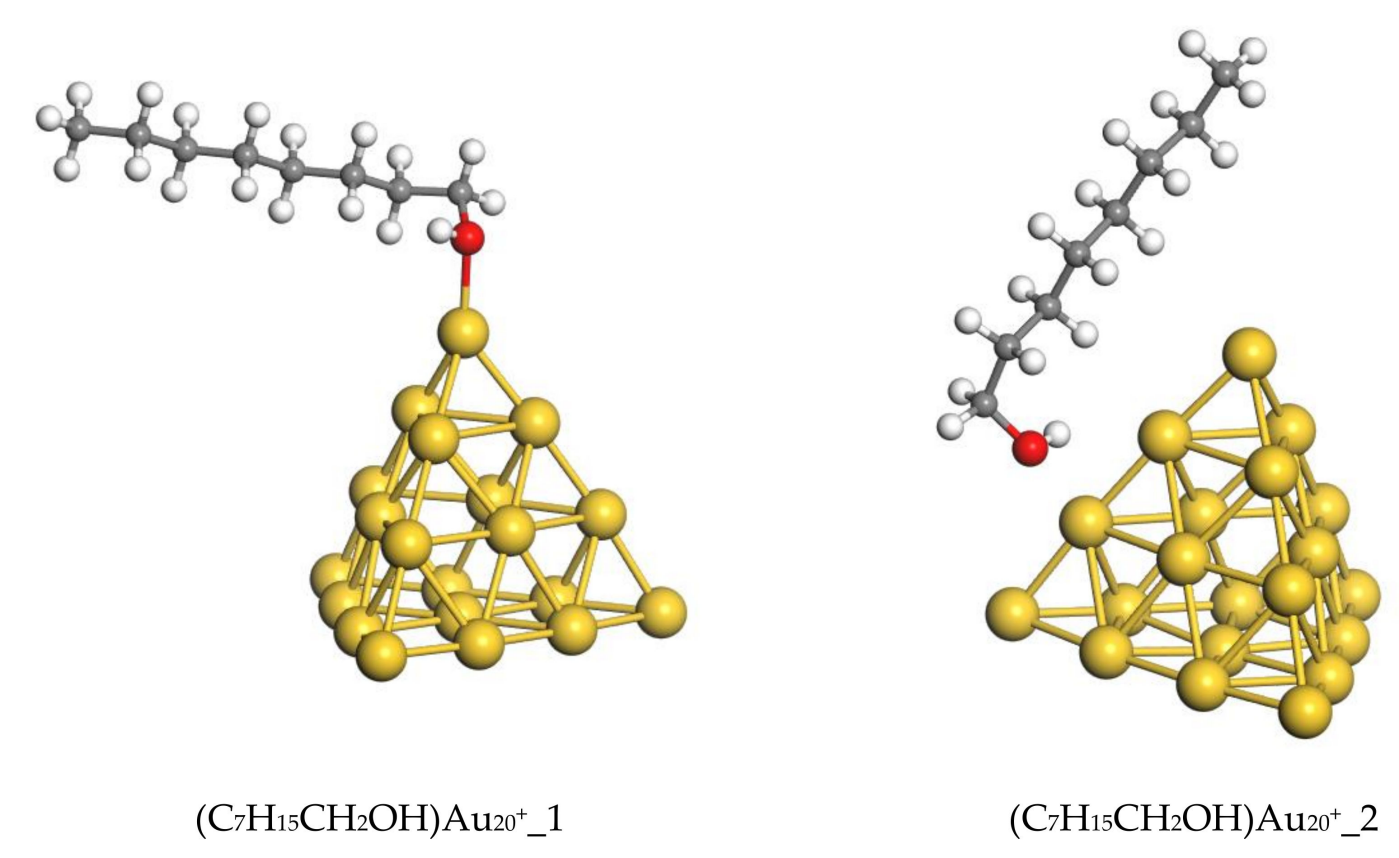
Optimized structures of *n*-octanol-Au_20_ and *n*-octanol-Au_20_^+^ complexes.

The optimized structures of octanol-Au_20_ complexes with alcohol coordination on different gold atoms are shown in Figure 7. The energy changes during the formation of the complexes and the corresponding standard enthalpies are collected in Table 7. The optimization of octanol-Au_20_ complex, in which alcohol is coordinated on the facet of the cluster, has led to the (C_7_H_15_CH_2_OH)Au_20__2 complex with edge coordination. The OH group is involved in the interaction of the alcohol and the cluster. The calculated Au–O distances are 0.243 nm and 0.338 nm in (C_7_H_15_CH_2_OH)Au_20__1 and (C_7_H_15_CH_2_OH)Au_20__2, respectively. According to the calculated data, the most favorable coordination of *n*-octanol on Au_20_ is at the cluster’s top. The binding energy of alcohol on the edge atoms is significantly lower.

Optimized structures of octanol-Au_20_^+^ have features similar to those of neutral octanol-Au_20_. In contrast, the calculated energy changes in Reaction (R2) (Table 7) are larger than in Reaction (R1). The binding energy of *n*-octanol with low-coordinated cationic gold atoms through OH-group is twice as much than that of low-coordinated gold atoms of neutral cluster. So, it can be concluded that the cationic sites play an important role in *n*-octanol activation on gold nanoparticles.

The adsorption of *n*-octanol on gold nanoparticles was simulated using DFT. It was shown that the low-coordinated gold atoms on the clusters top are the most active in the activation of alcohol. The binding energy of *n*-octanol with low-coordinated cationic gold atoms is sufficiently higher than on the neutral cluster. Based on the quantum chemistry, it can be concluded that the cationic sites play an important role in *n*-octanol adsorption.

### 3.5. Quantum Chemical Simulation of n-Heptane Adsorption on Au^0^, Au^+^ and Au^+3^

**Figure 8:**
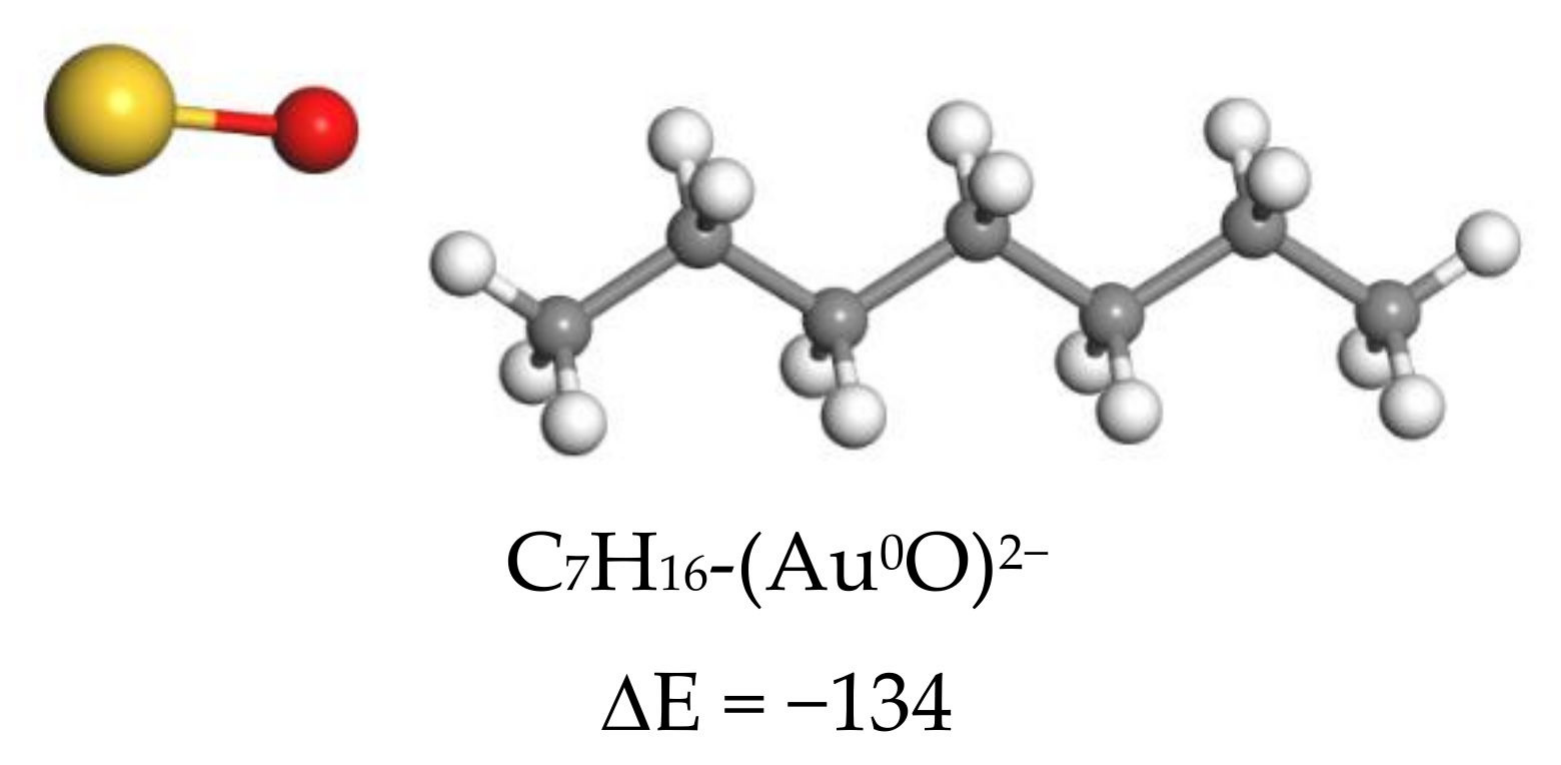

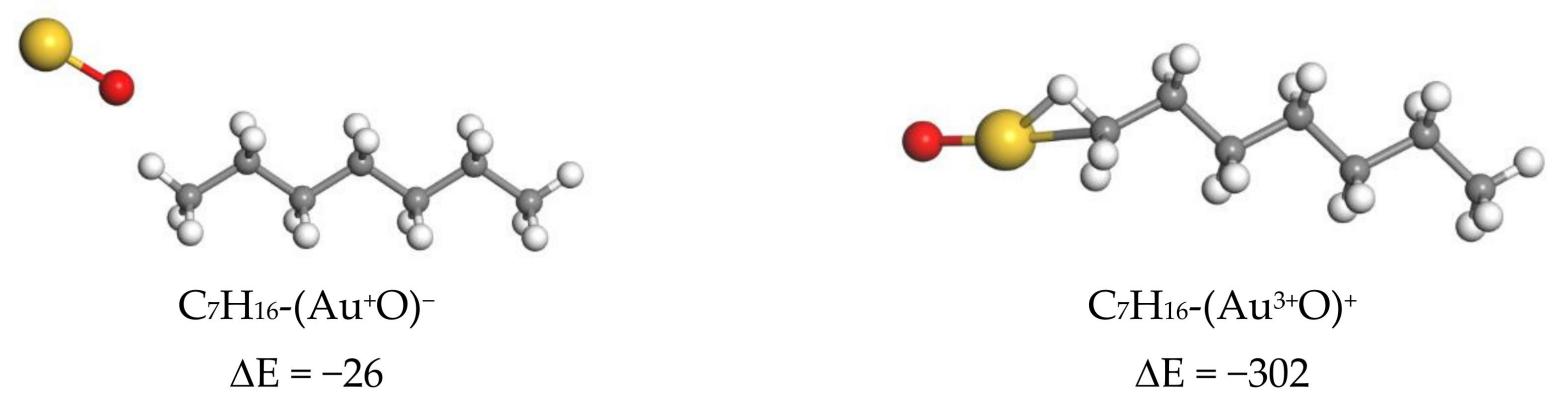
Optimized structures of C_7_H_16_-(Au^0^O)^2−^, C_7_H_16_-(Au^+^O)^−^ and C_7_H_16_-(Au^+3^O)^+^. Calculated adsorption energies are given in kJ/mol.

To reveal the role of Au^3+^ sites as inhibitors of the catalytic reaction, a quantum chemical simulation of the adsorption of the solvent (heptane) on simple models containing Au^0^, Au^+^ and Au^3+^ was performed. (Au^0^O)^2−^, (Au^+^O)^−^ and (Au^3+^O)^+^ molecules were considered as models. Optimized structures of C_7_H_16_-(Au^0^O)^2−^, C_7_H_16_-(Au^+^O)^−^ and C_7_H_16_-(Au^3+^O)^+^ complexes and calculated values of adsorption energies are presented in Figure 8.

Heptane can interact with (Au^0^O)^2−^ with an adsorption energy of −134 kJ/mol. This suggests that the Au^0^ sites may be partially occupied by solvent molecules. In contrast, heptane forms the most stable complex with (AuO)^+^, in which gold is in the trivalent state. The calculated adsorption energy in C_7_H_16_-(Au^3+^O)^+^ is −302 kJ/mol. Using small models of active sites containing Au^0^, Au^+^ and Au^3+^, it was shown that (Au^3+^O)^+^ cations can strongly bind heptane.

## 4. Conclusions

The efficiency of gold catalysts supported on titania modified with iron or magnesium oxides and different gold contents (0.5 or 4 wt.%), and different thermal pretreatment conditions (H_2_ or O_2_), were investigated in the aerobic oxidation of *n*-octanol under mild conditions.

It was found that the catalytic behavior of Au/M_x_O_y_/TiO_2_ systems is primarily determined by the electronic state of the deposited gold; namely, the ratio between the Au^0^, Au^+^ and Au^3+^ states, which is determined by the nature of the support and modifier, pretreatment conditions and gold concentration. A comparative study revealed that Au^+^ plays a decisive role in the oxidation of *n*-octanol and acts as an active site of Au/M_x_O_y_/TiO_2_ catalysts. Au^3+^ ions play an inhibitory role in the oxidation of *n*-octanol, due to the strong adsorption of the solvent and/or the blocking of highly charged trivalent gold ions by water molecules from the reaction. The low catalytic activity of the samples where only metallic gold was detected, according to the XPS and DRIFT CO data, was probably due to the partial blocking of the Au^0^ sites by the solvent that also led to inhibition of the oxidation of *n*-octanol, as in the case of Au^3+^.

Density functional theory (DFT) simulations confirmed that Au^+^ sites play an important role, while Au^0^ and mostly Au^3+^ has an inhibitory effect *n*-octanol oxidation. By varying the nature of the modifier, the pretreatment atmosphere and the gold content, it was possible to obtain the optimal ratio between Au^0^, Au^+^ and Au^3+^ states, thereby increasing the efficiency of Au/M_x_O_y_/TiO_2_ catalysts in the oxidation of *n*-octanol.

It was found that reaction product distribution, notably, the formation of acid and/or ester, depends on the ratio of Brønsted acid centers to Brønsted basic centers: with a high ratio no acid is formed; with a low ratio of BAC/BBC selectivity to acid higher then to ester; intermediate values of BAC/BBC ratio produce mixture of acid and ester. It should be mentioned that, such a trend in dependence selectivity on BAC/BBC ratio was not observed only for three catalysts with a high content of trivalent gold, where mainly the adsorption of solvent occurs.

The best results of *n*-octanol oxidation were achieved using modified samples with the lower gold content after oxidizing pretreatment at 300 °C for 1 h, namely 0.5% Au/MgO/TiO_2_ and 0.5% Au/Fe_2_O_3_/TiO_2_; reaching 43% and 41% conversion after 6 h, with the highest selectivity to ester in both cases.

## Figures and Tables

**Table 1:** Catalytic oxidation of *n*-octanol using gold catalysts under mild conditions (1 atm, 80 °C).

Catalyst	Au Content, wt.%	Oxidant	Solvent	Reaction time, h	R ^c^	Conv., %	Selectivity, %			Ref.
							Aldehyde	Acid	Ester	
Au/SiO_2_	6	O_2_	toluene	4	8	40	17			[24]
Au/Ga_3_Al_3_O_9_	1	O_2_	toluene	3	67	23	99			[25]
Au/MIL-101 ^a^	0.5	O_2_	toluene	3	67	38	99			[25]
Au/γ-Ga_2_O_3_	2.5	O_2_	Mes. ^d^	2.5	10	45	99			[26]
Au/Cu_a_Mg_b_Al_c_O_x_	1	O_2_	Mes.	3	95	34 ^e^	98			[27]
Au-Nb_2_O_5_-D ^b^	2	O_2_	*n*-heptane	6	100	5	58	0	42	[28]
Au-Nb_2_O_5_-H ^b^	2	O_2_	*n*-heptane	6	100	6	45	0	55	[28]
Au-ZnO-D	2	O_2_	*n*-heptane	6	100	45	18	0	82	[28]
Au-ZnO-H	2	O_2_	*n*-heptane	6	100	38	20	0	80	[28]
Au-MgO-D	2	O_2_	*n*-heptane	6	100	65	15	10	75	[28]
Au-MgO-H	2	O_2_	*n*-heptane	6	100	27	35	15	50	[28]
Au/TiO_2__pH_2_	4	O_2_	*n*-heptane	6	100	11	81	3	16	[29]
Au/CeO_2_/TiO_2__pH_2_	4	O_2_	*n*-heptane	6	100	23	66	2	32	[29]
Au/La_2_O_3_/TiO_2__pH_2_	4	O_2_	*n*-heptane	6	100	40	36	0	64	[29]
Au/Fe_2_O_3_/TiO_2__pH_2_	4	O_2_	*n*-heptane	6	100	15	58	0	42	[30]
Au/MgO/TiO_2__pH_2_	4	O_2_	*n*-heptane	6	100	20	67	2	31	[30]
Au/TiO_2__pO_2_	0.5	O_2_	*n*-heptane	6	100	17	28	52	20	[32]
Au/Fe_2_O_3_/TiO_2__pO_2_	0.5	O_2_	*n*-heptane	6	100	41	36	21	43	This
Au/MgO/TiO_2__pO_2_	0.5	O_2_	*n*-heptane	6	100	43	38	20	42	work
Au/CeO_2_/TiO_2__pO_2_	0.5	O_2_	*n*-heptane	6	100	30	27	43	30	[32]
Au/La_2_O_3_/TiO_2__pO_2_	0.5	O_2_	*n*-heptane	6	100	63	26	11	63	[32]
Au/TiO_2__pO_2_	0.5	TBHP ^f^	-	6	5000	27	0	80	20	[33]
Au/CeO_2_/TiO_2__pO_2_	0.5	TBHP	-	6	5000	30	0	80	20	[33]
Au/La_2_O_3_/TiO_2__pO_2_	0.5	TBHP	-	6	5000	58	0	80	20	[33]

^a^ MIL-101: zeolite-type metal-organic framework; ^b^ D, dried; H, after thermal reductive treatment; ^c^ R: alcohol/Au ratio (mol/mol); ^d^ mes.: mesitylene at 90 °C; ^e^ Instead of conversion, data on yield of aldehyde is given; ^f^ TBHP: *tert*-butyl hydroperoxide.

**Table 2:** Textural properties of supports and catalysts, analytical content and particle size of Au. Data for Entries 1–4 taken from [31,32].

Entry	Sample	S_BET_, m^2^/g		EDX Au Content, wt. %	Au Average Particle Size, nm
		Support	Catalyst		
1	0.5% Au/TiO_2__pH_2_	55	54	0.4	4.4
2	0.5% Au/TiO_2__pO_2_	55	54	0.4	4.2
3	4% Au/TiO_2__pH_2_	55	50	4.0	3.0
4	4% Au/TiO_2__pO_2_	55	50	4.0	3.3
5	0.5% Au/MgO/TiO_2__pH_2_	48	47	0.3	2.6
6	0.5% Au/MgO/TiO_2__pO_2_	48	47	0.3	3.2
7	4% Au/MgO/TiO_2__pH_2_	48	43	4.0	5.1
8	4% Au/MgO/TiO_2__pO_2_	48	43	4.0	2.9
9	0.5% Au/Fe_2_O_3_/TiO_2__pH_2_	49	49	0.5	3.1
10	0.5% Au/Fe_2_O_3_/TiO_2__pO_2_	49	49	0.5	3.4
11	4% Au/Fe_2_O_3_/TiO_2__pH_2_	49	44	3.2	5.2
12	4% Au/Fe_2_O_3_/TiO_2__pO_2_	49	44	3.2	3.2

**Table 3:** Effect of gold content (0.5 or 4 wt.%) and redox treatment (H_2_ or O_2_) on the different electronic states of Au (determined by XPS), and their catalytic activity for *n*-octanol oxidation for Au/TiO_2_, Au/Fe_2_O_3_/TiO_2_, Au/MgO/TiO_2_ catalysts. Entries 1–4 data are taken from [31,32].

Entry	Catalyst	Au Electronic State Relative Content, %			Conversion of *n*-Octanol after 6 h, %
		Au^0^	Au^+^	Au^3+^	
1	0.5% Au/TiO_2__pH_2_	91	9	0	3
2	0.5% Au/TiO_2__pO_2_	84	16	0	17
3	4% Au/TiO_2__pH_2_	73	14	11	11
4	4% Au/TiO_2__pO_2_	89	11	0	6
5	0.5% Au/MgO/TiO_2__pH_2_	81	11	8	12
6	0.5% Au/MgO/TiO_2__pO_2_	67	25	8	43
7	4% Au/MgO/TiO_2__pH_2_	51	29	20	20
8	4% Au/MgO/TiO_2__pO_2_	100	0	0	3
9	0.5% Au/Fe_2_O_3_/TiO_2__pH_2_	28	29	43	7
10	0.5% Au/Fe_2_O_3_/TiO_2__pO_2_	26	64	10	41
11	4% Au/Fe_2_O_3_/TiO_2__pH_2_	42	37	21	15
12	4% Au/Fe_2_O_3_/TiO_2__pO_2_	100	0	0	5

**Table 4:** Acid properties of catalysts.

Entry	Catalyst	Concentration of Acid Centers, µmol/g			
		Weak	Medium	Strong	Total Amount
1	0.5% Au/TiO_2__pH_2_	100	313	110	523
2	0.5% Au/TiO_2__pO_2_	167	250	337	754
3	4% Au/TiO_2__pH_2_	264	81	8	353
4	4% Au/TiO_2__pO_2_	260	66	9	335
5	0.5% Au/Fe_2_O_3_/TiO_2__pH_2_	86	114	41	241
6	0.5% Au/Fe_2_O_3_/TiO_2__pO_2_	189	440	234	863
7	4% Au/Fe_2_O_3_/TiO_2__pH_2_	298		54	352
8	4% Au/Fe_2_O_3_/TiO_2__pO_2_	176	398	320	894
9	0.5% Au/MgO/TiO_2__pH_2_	108	69	17	194
10	0.5% Au/MgO/TiO_2__pO_2_	203	177	36	415
11	4% Au/MgO/TiO_2__pH_2_	262		65	327
12	4% Au/MgO/TiO_2__pO_2_	369		67	436

**Table 5:** Basic properties of catalysts.

Entry	Catalyst	Concentration of Basic Centers, µmol/g				
		Weak		Medium	Strong	Total Amount
1	0.5% Au/TiO_2__pH_2_	46		66	15	127
2	0.5% Au/TiO_2__pO_2_	56		20	13	89
3	4% Au/TiO_2__pH_2_	15		41	17	73
4	4% Au/TiO_2__pO_2_	34		29	21	84
5	0.5% Au/Fe_2_O_3_/TiO_2__pH_2_	22	121	130	21	22
6	0.5% Au/Fe_2_O_3_/TiO_2__pO_2_	37		91	47	175
7	4% Au/Fe_2_O_3_/TiO_2__pH_2_	33	138	180	225	33
8	4% Au/Fe_2_O_3_/TiO_2__pO_2_	20		74	30	124
9	0.5% Au/MgO/TiO_2__pH_2_	23	163	126	32	23
10	0.5% Au/MgO/TiO_2__pO_2_	44		38	26	108
11	4% Au/MgO/TiO_2__pH_2_	23	78	66	69	23
12	4% Au/MgO/TiO_2__pO_2_	51		41	21	113

**Table 6:** Acid-basic properties of catalysts and corresponding selectivity data in *n*-octanol oxidation.

Entry	Catalyst	Concentration of Weak Centers, µmol/g			Selectivity after 6 h, %	
		Basic		Acid	Ester	Acid
1	0.5% Au/TiO_2__H_2_	46		100	12	39
2	0.5% Au/TiO_2__O_2_	56		167	20	52
3	4% Au/TiO_2__H_2_	15		264	16	3
4	4% Au/TiO_2__O_2_	34		260	42	3
5	0.5% Au/Fe_2_O_3_/TiO_2__H_2_	22	121	86	17	29
6	0.5% Au/Fe_2_O_3_/TiO_2__O_2_	37		189	43	21
7	4% Au/Fe_2_O_3_/TiO_2__H_2_	33	138	298	42	0
8	4% Au/Fe_2_O_3_/TiO_2__O_2_	20		176	51	1
9	0.5%Au/MgO/TiO_2__H_2_	23	163	108	16	53
10	0.5%Au/MgO/TiO_2__O_2_	44		203	42	20
11	4%Au/MgO/TiO_2__H_2_	23	78	262	31	2
12	4%Au/MgO/TiO_2__O_2_	51		369	42	0

**Table 7:** Calculated energy change (ΔE in kJ/mol) and standard enthalpy (ΔH, kJ/mol) in the Reactions (R1) and (R2) of *n*-octanol with Au_20_^z^ cluster (z = 0, +1).

z	Complex	Type of Coordination	ΔE_1_, ΔE_2_	ΔH_1_, ΔH_2_
0	(C_7_H_15_CH_2_OH)Au_20__1	top	−40	−36
0	(C_7_H_15_CH_2_OH)Au_20__2	edge	−23	−20
+1	(C_7_H_15_CH_2_OH)Au_20_^+^_1	top	−81	−78
+1	(C_7_H_15_CH_2_OH)Au_20_^+^_2	edge	−55	−51
